# High resolution structures define divergent and convergent mechanisms of archaeal proteasome activation

**DOI:** 10.1038/s42003-023-05123-3

**Published:** 2023-07-15

**Authors:** Janelle J. Y. Chuah, Matthew S. Rexroad, David M. Smith

**Affiliations:** 1 Department of Biochemistry and Molecular Medicine, West Virginia University School of Medicine, 64 Medical Center Dr., Morgantown, WV USA; 2 Department of Neuroscience, Rockefeller Neuroscience Institute, West Virginia University, Morgantown, WV USA

**Keywords:** Cryoelectron microscopy, Proteasome, Molecular medicine, Enzyme mechanisms

## Abstract

Considering the link between neurodegenerative diseases and impaired proteasome function, and the neuro-protective impact of enhanced proteasome activity in animal models, it’s crucial to understand proteasome activation mechanisms. A hydrophobic-tyrosine-any residue (HbYX) motif on the C-termini of proteasome-activating complexes independently triggers gate-opening of the 20S core particle for protein degradation; however, the causal allosteric mechanism remains unclear. Our study employs a structurally irreducible dipeptide HbYX mimetic to investigate the allosteric mechanism of gate-opening in the archaeal proteasome. High-resolution cryo-EM structures pinpoint vital residues and conformational changes in the proteasome α-subunit implicated in HbYX-dependent activation. Using point mutations, we simulated the HbYX-bound state, providing support for our mechanistic model. We discerned four main mechanistic elements triggering gate-opening: 1) back-loop rearrangement adjacent to K66, 2) intra- and inter- α subunit conformational changes, 3) occupancy of the hydrophobic pocket, and 4) a highly conserved isoleucine-threonine pair in the 20S channel stabilizing the open and closed states, termed the "IT switch." Comparison of different complexes unveiled convergent and divergent mechanism of 20S gate-opening among HbYX-dependent and independent activators. This study delivers a detailed molecular model for HbYX-dependent 20S gate-opening, enabling the development of small molecule proteasome activators that hold promise to treat neurodegenerative diseases.

## Abstract (web summary)

Cryo-EM structures and mutagenesis of the archaeal 20S proteasome with a small molecular activator mimicking the HbYX motif reveal new details of a conserved gating switch mechanism and mechanistic differences between HbYX-dependent and -independent gate-opening

## Introduction

The ubiquitin-proteasome system (UPS) is a crucial regulatory pathway responsible for eliminating damaged or unnecessary proteins within cells^1^. Central to this system is the proteasome, a molecular machine that selectively degrades proteins. Proteasome activators (PAs) play key roles in regulating this process to enable and ensure selective protein degradation. Dysregulation of the proteasome is associated with neurodegenerative diseases (NDs), characterized by impairment of proteasome function^2–7^.

The eukaryotic proteasome’s core particle, known as the 20S, comprises four stacked heteroheptameric rings (α-β-β-α) with a central substrate entry pore at each end. The gate, mainly formed by the N-terminus of α2, α3, and α4, regulates substrate entry by closing off the barrel-shaped structure of the 20S^8^. The closed gate conformation obstructs the central pore, preventing protein entry for degradation. The N-terminus of each α subunit carries a YDR (tyrosine-aspartic acid-arginine) motif that interacts with neighboring N-termini to stabilize the closed gate state^8^. These N-termini extensions can adopt an “open” state, where they point outwards from the α ring pore, stabilized by an alternative interaction from the YDR motif^9^. Truncation of α3’s N-terminus (α3∆N), which acts as a central lynchpin to stabilize the closed state, generates a constitutively open (active) 20S that efficiently degrade unstructured proteins^8,10^.

The gate-opening process in the 20S can be induced by the binding of proteasome regulatory complexes, which have been described to use one of two gate-opening mechanisms, HbYX-dependent or 11 S family-dependent^11,12^. Arguably, the most frequently studied HbYX-dependent proteasome activator is the 19S, also known as PA700 or the Regulatory Particle (RP), which associates with the 20S to form the 26S complex that degrades ubiquitinated proteins. The 19S consists of a base subcomplex, which is primarily composed of a heterohexameric ring of ATPases (Rpt1-6), and a lid subcomplex, which contains ubiquitin binding and processing subunits. The 19S has been shown to stimulate gate-opening by the docking of the C-terminal tails of Rpt1-6, some of which contain the HbYX motif, in the intersubunit pockets of the 20S α ring^13^. Other PA’s also associate with the 20S via their C-termini such as PA200/Blm10 and the 11S activators.

The HbYX motif located at the C-terminus is universally conserved across all organisms with proteasomes, which includes all archaea and eukaryotes. This motif was first recognized in PAN (Proteasome-Activating Nucleotidase), an archaeal homolog of the 19S ATPases. The architecture of the archaeal 20S proteasome is conserved resembling the eukaryotic 20S, but its α and β rings are homoheptameric rather than heteroheptameric. Furthermore, the YDR motif is conserved in the archaeal 20S, and its gate also transition between closed and open states^14^. While its open-gate structure is similar to eukaryotic 20S, the closed-gate structure in archaea is different due to homomeric nature. The C-terminal HbYX motif of PAN also binds similarly to the intersubunit pockets in the archaeal 20S in a conserved fashion^13,15^.

Previous research demonstrate that HbYX-dependent proteasome activators in both eukaryotes and archaea use the HbYX motif not only to bind to the 20S but also to induce gate-opening, unlike the 11 S family^13,15^. In fact, peptides corresponding to the C-terminus of PA26 (a member of the 11 S family) cannot induce gate-opening autonomously^15^, while peptides corresponding to the C-terminus of Rpt2, Rpt3, Rpt5, PAN, and PA200/Blm10 can induce gate opening by them selves^15,16^. The C-terminal HbYX motif binds into pockets formed by the interface of the α subunits in the 20S, called intersubunit pockets^13,15^. Interestingly, structures of the 26S suggest that not all intersubunit pockets need to interact with the motif to induce gate opening, as only the C-terminus of Rpt2, 3, & 5 from the 19S heteromeric ATPases have the HbYX motif while Rpt1 has a partial HbYX motif, lacking the Hb residue. The roles that the C-termini of Rpt4 and Rpt6 (which lack the HbYX motif) play in the association of the 19S-20S and 20S gating regulation are unclear^17–19^. Several prior studies suggest that the binding of HbYX-peptides to intersubunit pockets, structurally distant from the gating residues, results in proteasome activation due to gate opening^13,15,16,20–23^.

The 11 S family constitute heptameric complexes that lack the HbYX motif and rely on an array of “activation loops” that interface directly with the base of the gating N-termini in the pore of the α ring^24,25^. Structural studies suggest that minimal conformational changes in the α subunits (excluding gating regions) are necessary for gate-opening by the 11 S activators^9^ (e.g., PA26). These activation loops appear to sterically repel a reverse turn proline (Pro17) at the base of the gating residues shifting it by <1 Å, which is sufficient to disrupt the closed state and stabilize the open state^9^. Evidently, the two families of PAs (HbYX-dependent and HbYX-independent) use similar but different strategies to induce 20S gate-opening. Although the location and effect of HbYX-binding has been investigated, the molecular mechanism of HbYX-dependent gate opening appears to be surprisingly complex and remains unsolved. Several structural studies have been done using PA26 with modified activation loops, and added HbYX motifs to their C-termini^22,23,26^, but these have obvious limitations to rigorously distinguish HbYX-dependent and HbYX-independent (11 S) mechanisms.

Structural studies of human and yeast 26S (H26S and Y26S, respectively) proteasome have shown variations in the binding patterns of C-terminal tails of the ATPases during gate-opening. As the H26S transition towards a more active state (E_A1_,_2_ > E_B_ > E_C1,2_ > E_D1,2_)^17^, more C-termini form stable interactions (as observed via cryo-EM^17^), starting with Rpt3, Rpt5 and Rpt2, then Rpt6, and finally Rpt1. Structures show the first tails to dock (Rpt3, 5, & 2) all carry the HbYX motif, yet the gate does not appear open. When the last C-terminus of the ATPases binds (Rpt1, which has a partial HbYX motif), a conformational change occurs, resulting in a stably opened gate. While another structural study of Y26S^27^ suggests the same pattern of C-terminal tail binding for gate opening, other studies on the Y26S^18,28^ suggest complete gate-opening occurs after the binding of Rpt6. In other words, the binding of Rpt1 in the α ring is not required for complete gate-opening, at least in Y26S. Moreover, the same Y26S structures^18,28^ indicate that the binding of Rpt2, 3, & 5 alone is sufficient to trigger partial gate-opening. While cryo-EM findings can approximate gate openness^29^, the structures do not precisely reflect the dynamics of the gate within an individual proteasomal state (E_A1_,_2,_ E_B,_ E_C1,2_, E_D1,2_). For example, the un-activated 20S proteasome by itself can still degrade linearized proteins or peptides, but structurally, it is observed with a closed gate via cryo-EM or X-ray crystallography, suggesting that there is a limit to the conclusions that can be drawn regarding the dynamics of the gate from these structural biology techniques.

While the molecular interactions that stabilize the closed and fully open state of the proteasome’s gate are well-studied^8,30^, as are the 26S proteasome opened/closed states^17,18,27,28^, the complex molecular mechanisms that allosterically regulate the transition between these closed and open states are not understood. Moreover, a clear understanding of the HbYX-dependent gate-opening mechanism in the 20S will provide the molecular framework to guide drug-discovery approaches aimed at activating proteasomal degradation to treat ND.

This study presents a mechanistic model for the activation of the proteasome core particle by the HbYX (hydrophobic-tyrosine-variable C-terminal residue)-motif, that is found on most PA’s. We employ a small molecule developed in a separate but not unrelated study^31^, namely ZYA, that functionally emulates the HbYX-dependent mechanism of proteasomal gate-opening in archaeal and eukaryotic proteasomes as a model for the HbYX motif. Our high-resolution cryo-EM structure of the archaeal proteasome in complex with the small molecule activator, compared to other structures of our own and one previously published by Hill and colleagues, provides mechanistic insights into the HbYX-dependent proteasome gate-opening, which is conserved from archaea to humans. Our major findings include three mechanistic features of proteasomal gating: (1) a HbYX-induced rearrangement of the loop on the outer edge of the 20S, which could be a target of neurodegenerative disease-related oligomers, (2) inter- and intrasubunit conformational changes caused by HbYX-binding, and (3) a gating switch mechanism found to be relevant on all proteasome activators. More importantly, these findings uncovered in archaeal 20S are also conserved in the human 20S, advancing our general mechanistic understanding of proteasome gate-opening and thus its activation for protein degradation.

## Results

### Structure of ZYA-bound archaeal proteasome shows global conformational changes, particularly in the α ring

**Fig. 1:**
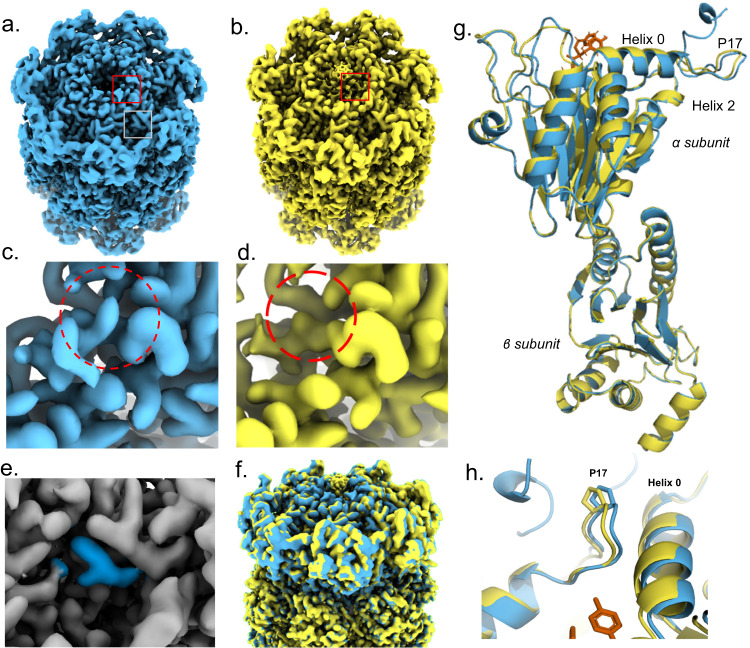
Structure of ZYA bound to the T20S proteasome (1.9 Å) showing global conformational changes in the α ring. **a** ZYA-T20S unsharpened electron density map (blue; 1.9 Å); red square highlights the view in **c**; gray square highlights the view in **e**. **b** WT T20S unsharpened electron density map (yellow; 2.06 Å); red square highlights the view in **d**. **c** ZYA-T20S electron density map (blue) of red box region in a with red dotted circle highlighting density corresponding to tyrosine of YDR motif in the gate-open state. **d** WT T20S electron density map (yellow) of boxed region in **b**, with red dotted circle highlighting location of the YDR motifs tyrosine in the open state. Absence of density in this location indicates a “closed” state, which is confirmed by densities, at lower map threshold levels, near the center of the α ring (seen in **b**). **e** ZYA-T20S electron density map (gray) of α intersubunit pocket (gray box region in **a**), with densities corresponding to bound ZYA ligand (blue). **f** Overlay of WT (yellow) and ZYA-T20S (blue) electron density map, showing the α and β rings, aligned by the β ring. **g** Overlay of WT (yellow) and ZYA-T20S (blue) α and β subunits models, aligned by β subunit. **h** Close-up on Helix 0 and Pro17, shown in sticks, aligned by β subunit to demonstrate intersubunit conformational changes.

In a separate study^31^ we showed ZYA, a peptide mimetic that emulates the HbYX motif, robustly induces gate opening in archaeal, yeast and mammalian 20S proteasomes demonstrating a conserved activation mechanism. To determine the details of how the HbYX motif induces gate-opening in the archaeal 20S proteasome, we used 20S from *Thermoplasma acidophilum* (T20S) and incubated it with ZYA. An advantage of determining the ZYA-20S structure in the T20S is that it is D7 symmetric, allowing for D7 symmetry application during reconstruction, which maximizes the achievable resolution and concomitant molecular details. Here, using cryo-EM, we generated a 1.9 Å structure of ZYA bound to T20S (ZYA-T20S; PDB:8F7K) (Fig. 1a; Supplementary Figs. 1, 2) and a 2.1 Å WT T20S structure (PDB: 8F6A) (Fig. 1b; Supplementary Figs. 3, 4) (Table 1).1b; Supplementary Figs. 3, 4). Comparing against our WT T20S structure, we noted strong densities in our ZYA-T20S map, corresponding to the YDR region (Fig. 1c, d). Additionally, we noted a lack of density in the central channel showing the N-termini pointing up in the ZYA-T20S map, compared to the WT T20S map, clearly indicating a conformation that corresponds to the open-gate state (Fig. 1a, b). This ZYA-T20S structure resolves the N-termini of the α subunits up to Gly4, which include three N-terminal residues that have not been previously resolved. The WT T20S map does show clear pore-central densities that are expected for the closed gate (Fig. 1b). In addition, our WT map also resolves the N-termini up to Ala11, which includes 2 additional residues not previously resolved in the closed state. In the ZYA-T20S map, densities corresponding to ZYA bound to the α intersubunit pockets were clearly visible (Fig. 1e, in blue) and resembled the expected structure of this dipeptide. Alignment of the β-rings and subunit between the maps and models (Fig. 1f, g) indicate clear intersubunit conformational changes in the α ring (no significant changes were seen in the β subunits). These intersubunit conformation changes are presented as a rotation of the α-ring (Supplementary Movie 1). This apparent rotation in the α-ring is primarily due to individual rigid body movements of each α-subunit (Fig. 1f, g), centered around the length of Helix 2, which acts as the pivot. Helices’ 3, 4, and 5, which are most distant from the pivot move by ~2 Å.

**Table 1:** Cryo-EM data collection, refinement, and validation statistics.

	T20S-ZYA EMDB-28906 PDB 8F7K	WT T20S EMDB-28878 PDB 8F6A	T20S L81Y EMDB-28876 PDB 8F66
Data collection and processing			
Magnification	×81,000	×81,000	×81,000
Voltage (kV)	300	300	300
Electron exposure (e–/Å^2^)	50	50	50
Defocus range (μm)	−2.4 to −1.2	−2.4 to −1.2	−2.4 to −1.2
Pixel size (Å)	0.54	0.54	0.54
Symmetry imposed	D7	D7	D7
Initial particle images (no.)	1,079,760	940,560	1,352,216
Final particle images (no.)	871,770	444,678	131,453
Map resolution (Å)	1.94	2.06	2.28
FSC threshold	0.143	0.143	0.143
Map resolution range (Å)	1.73–26.10	1.86–27.00	2.07–30.47
Refinement			
Initial model used (PDB code)	1YA7	1YA7	1YA7
Model resolution (Å)	1.9	2.0	2.2
FSC threshold	0.143	0.143	0.143
Model composition			
Non-hydrogen atoms	47,264	45,948	46,508
Protein residues	6048	5950	6006
Ligands	14	0	0
*B* factors (Å^2^)	(min/max/mean)	(min/max/mean)	(min/max/mean)
Protein	1.67/78.10/17.90	0.95/69.94/13.96	1.19/84.10/16.39
Ligand	0.50/0.50/0.50		
R.m.s. deviations			
Bond lengths (Å)	0.005	0.004	0.005
Bond angles (°)	0.685	0.693	1.019
Validation			
MolProbity score	1.22	1.76	2.14
Clashscore	3.21	4.08	6.86
Poor rotamers (%)	1.39	3.21	3.94
Ramachandran plot			
Favored (%)	98.6	96.91	95.53
Allowed (%)	1.40	3.09	3.76
Disallowed (%)	0	0	0.71
Ramachandran *Z* score			
Whole	1.42	1.20	0.82
Helix	2.03	2.27	1.33
Sheet	0.83	0.48	0.72
Loop	−0.06	−0.62	−0.47

**Fig. 2:**
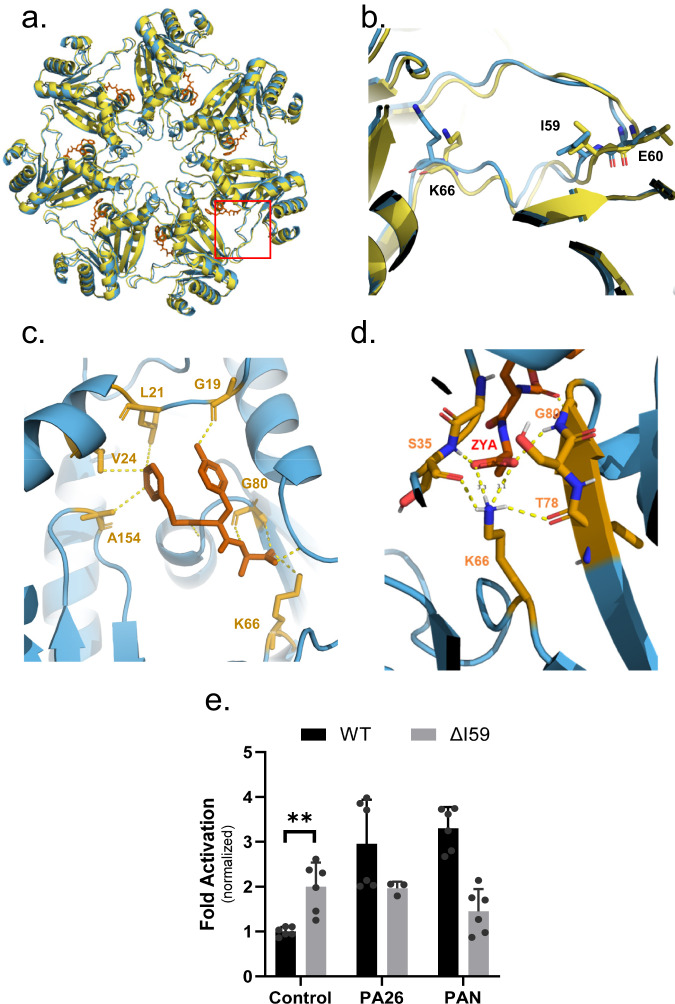
ZYA binds to the T20S intersunit α pockets, triggering rearrangement of Lys66 and the pocket “back-loop” which is involved in gate-opening. **a** Top view of the atomic model of WT T20S (yellow) and ZYA-T20S (blue) overlayed after alignment of β subunit rings. Showing only the α subunits here for clarity. **b** View of loop proximal to K66 from red box in a, with key residues discussed shown in sticks, aligned by α subunit to demonstrate intrasubunit conformational changes. Similar changes are seen in β alignments. **c** ZYA (red) in the α intersubunit pocket, showing key interactions with selected α subunit residues (orange). **d** A network of ionic interactions between ZYA’s C-terminal carboxy, K66 side chain, and indicated residues in the α-intersubunit pockets that stabilize the shifted state of K66 that can be seen in **a**. **e** 7 nM T20S (WT or ∆I59) incubated with 44 nM PA26 or 15 nM PAN (supplemented with ATP and MgCl_2_). LFP degradation rate (rfu/min) normalized to control and the amount of proteasome from different preparations is normalized to their rate of LLVY-AMC hydrolysis, which is insensitive to gating affects (see methods for details). Data (means) are representative of three or more independent experiments each performed in triplicate. Error bars represent ± standard deviation.

In contrast, intrasubunit conformational changes were assessed by aligning a single α subunit from WT T20S and T20S ZYA structures. Though subtle, these intrasubunit changes were present. The Pro17 loop and connected N-terminal extension were shifted by ~1.0 Å in a direction perpendicular to Helix 0 (Fig. 1h), for comparison, the intersubunit change of Pro17 is 1.3 Å so most of the Pro17 shift comes from intrasubunit changes. Even Helix 0 shifted by about ~0.6 Å (1.0 A for intersubunit changes) moving in the direction pointed towards the Pro17 loop (Fig. 1h). These subtle intersubunit changes were also clearly visible in the electron density map (Fig. 1f). In addition, intersubunit conformational change was observed in the loop (S50-E65; back-loop), which is in the outer portion of the intersubunit pocket, adjacent to K66 (Fig. 2a, b). Though the local resolution of this loop is around 2.7 Å, it clearly changes conformation, with the bottom of the loop from I59 to K66 moving in the direction of K66 anywhere from ~1–2 Å. This motion appears to be causing or accommodating the rotation of the α subunits described above. We conclude that binding of the HbYX-like ZYA molecule causes unique inter- and intrasubunit conformational changes that allosterically switch the T20S from the closed to the open state.

Next, we analyzed the HbYX dipeptide’s interactions with the intersubunit pocket to deduce its mechanism for activating proteasome gate-opening (Fig. 2c). The carboxybenzyl group (i.e., Hb group) docks in a hydrophobic pocket, interacting with V24, L21, and A154, 3.7 Å, 4.3 Å and 3.6 Å away, respectively (Fig. 2c). The hydroxyl group of ZYA’s tyrosine hydrogen bonds with the backbone of G19 (Fig. 2c), sandwiched between L81 and K33, as would be expected for the penultimate tyrosine of the HbYX motif^13,22,23^. Additionally, we noted the backbone of the dipeptide hydrogen bonding with the backbone of G80, V82, and ZYA’s C-terminal carboxylic group forming a salt bridge with the sidechain of K66 (Fig. 2c), consistent with previous observations on the importance of K66 for HbYX-dependent gate-opening^9,15,26^. These noted interactions indicate that ZYA binds identically as shown for other HbYX motifs.

The ZYA-T20S model also showed that the binding of ZYA to the intersubunit pocket shortens the distance between G19 on one subunit and K66 on the neighboring subunit by ~1 Å (essentially the walls of the intersubunit pocket are pulled together in the range of 1-2 Å), primarily due to a shift of K66 α carbon (Fig. 2b, c). Based on these interactions and changes in the pocket, we deduce that ZYA binding acts as a “cable” that bridges across the intersubunit pocket connecting Helix 0 of an α subunit to the K66 loop in the neighboring subunit. The length of this “cable” is just short enough to “pull” or shift the K66 towards its new position (Fig. 2b, c). The result of displacing this K66 appears to be a rearrangement of the adjacent back-loop, resulting in the end of the loop (e.g., L57) moving in a direction away from the neighboring subunit and towards K66 by ~1.2 Å. In addition, the neighboring subunit Helix 3 follows the K66 loop to cause or accommodate the rigid body rotation of the α subunit (Supplementary Movie 1). Thus, the intersubunit “bridging” role ZYA plays leads to a K66 shift that promotes a rigid body rotation of the α subunits. Interestingly, the rigid body rotation and intrasubunit shifts in Helix 0 combine to shift Helix 0 away from the Pro17 that is in the neighboring subunit (Supplementary Movie 1). Since the base of Pro17 packs against Helix 0 in the neighbor, and Pro17 is on a flexible loop, it is able to move with its neighboring Helix 0 causing it to shift ~1.1 Å, which is known to be associated with gate-opening.

In addition to the above-mentioned ionic interactions, we also noticed the K66 and ZYA carboxy group participate in a highly coordinated network of H-bonds due to the new position that K66 takes after ZYA binding. In fact, in addition to the K66 interaction, the carboxy group of ZYA also interacts with the backbone of G80 and S35, flanking both sides of the carboxy-K66 salt bridge (Fig. 2d). Moreover, Lys66 also H-bonds to the backbone of S35 and T78, flanking both sides of the salt bridge. It appears that this network of six distinct ionic interaction stabilizes the K66 in this new position, likely stabilizing the rearrangement of the K66 adjacent back-loop. Since ZYA binding shifted the back-loop proximal to K66 (Fig. 2a, b) towards K66, so we asked whether shortening the loop by a single residue deletion affects gating. The deletion of I59 (∆I59) (Fig. 2e) resulted in slightly higher 20S activity (p-value: 0.0013), e.g., a more open gate (Fig. 2e). In addition, neither PAN (a HbYX-dependent activator) nor PA26 (non-HbYX-dependent activator) could stimulate the activity of T20S-∆I59 (Fig. 2e). This mutation suggests that the loop proximal to K66 does affect gating as expected, demonstrating that this K66 back-loop is important for regulating gate-function.

### Mutations that introduce aromatic rings in the Hb binding pocket induce gate opening

**Fig. 3:**
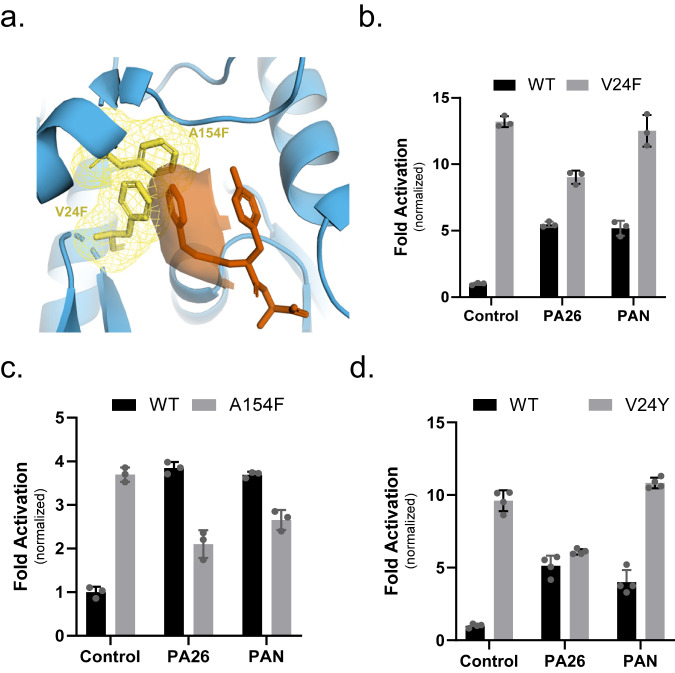
Mutations that introduce aromatic rings into the Hb (Z) binding pocket induce gate opening. **a** Structure of ZYA-T20S with simulated mutation of A154F and V24F shown in yellow sticks with dotted clouds, showing overlap between these mutants and the Z group binding location (ZYA; colored in red). **b** 7 nM T20S (WT or V24F) incubated with 44 nM PA26 or 15 nM PAN (supplemented with ATP and MgCl_2_). LFP degradation rate (rfu/min) normalized to control and the amount of proteasome from different preparations is normalized to their rate of LLVY-AMC hydrolysis, which is insensitive to gating affects (see methods for details). **c** Same as b but 7 nM T20S (WT or A154F). LFP degradation rate (rfu/min) normalized as in **b**. **d** Same as **b** but 7 nM T20S (WT or V24Y). LFP degradation rate (rfu/min) normalized as in **b**. Data (means) are representative of three or more independent experiments each performed in triplicate. Error bars represent ± standard deviation.

Next, we asked what role the Hb (i.e., Z) group on ZYA plays in gate opening, does it contribute to affinity, or does it actively play a role in inducing gate opening. To answer these questions, we mutated V24 and A154 (both in the Hb binding pocket) to phenylalanine, emulating the binding of the Z (benzene) in the pocket. Mutagenesis of these two residues to phenylalanine in Pymol shows they would occupy overlapping space with the Z group of ZYA (Fig. 3a). Both T20S variants, V24F and A154F, did in fact have a far higher activity than the WT control (Fig. 3b, c), with V24F being the most activating (~14-fold). Additionally, PAN and PA26 could neither further stimulate V24F and A154F mutants (Fig. 3b, c). This could be because the gate could not be further opened, or it could be that altering the Hb binding sites prevents them from binding to the 20S. Since V24F stimulated gate opening so strongly, even stronger than WT with saturating PA26, this suggests that introducing a large aromatic group in the Hb binding pocket by itself is sufficient to cause maximal gate opening. To test this hypothesis, we generated a V24Y variant, mutating V24 to another residue with a large aromatic group that is also observed to be in the Hb position of the HbYX motif on some proteasome activators (e.g., human Rpt5, yeast Blm10, mammalian PA200). As hypothesized, V24Y T20S had higher activity than the WT control and PAN and PA26 could not further stimulate (Fig. 3d). Interestingly, peptide degradation by T20S-V24F, A154F, and V24Y is reduced when PA26 is added, compared to controls. These results suggest that presence of a PA bound to the fully open-T20S slows the rate of peptide entry into the proteasome. We expect that the internal channel-loops in PA26 reduce the substrate diffusion rate relative to the fully open-T20S causing this reduced activity. In addition, PAN appears to slightly reduce activity of the A154F mutation. Proteasome ATPase inhibition of substrate entry has also been observed in the E_a_/E_b_ states of the 26S proteasome, while the E_d_ state is stimulated^17,27^. Other more complicated allosteric contributions could also be at play. These indicate that the Z group of ZYA likely plays an important and direct role in ZYA’s mechanism of action.

### Water molecules that interact with ZYA and the intersubunit pockets suggested to be important in gating

**Fig. 4:**
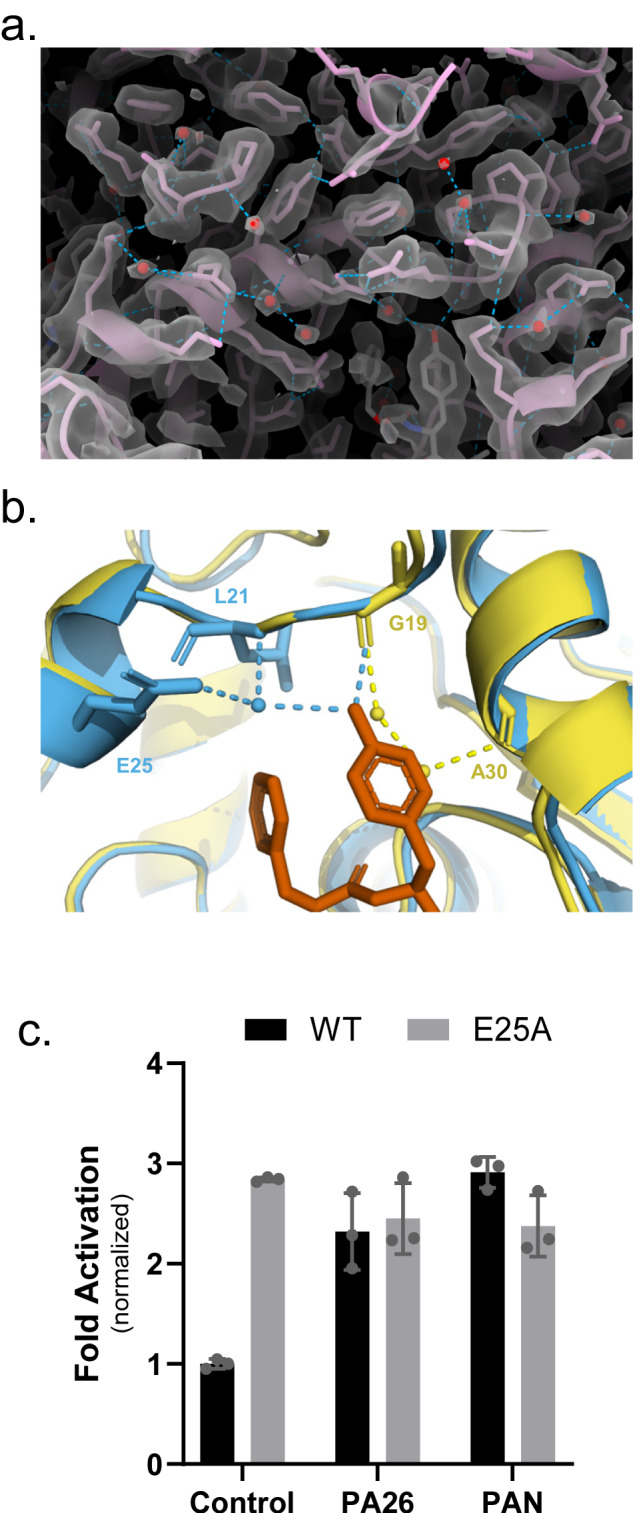
Water molecules that interact with ZYA and the intersubunit pocket residues appear to be important for gating regulation. **a** Overlay of ZYA-T20S electron density map (transparent surface) and model (pink sticks), showing waters (red spheres) modeled into the map and interactions (blue dotted lines) with the model. **b** Model of ZYA (red) docked in α intersubunit pocket, aligned by β subunits, showing interactions with waters (spheres) and residues (sticks) in the ZYA-T20S model (blue). ZYA-T20S model is overlapped with doused WT T20S model (yellow) for comparison. **c** 7 nM T20S (WT or E25A) incubated with 44 nM PA26 or 15 nM PAN (supplemented with ATP and MgCl_2_). LFP degradation rate (rfu/min) normalized to control and the amount of proteasome from different preparations is normalized to their rate of LLVY-AMC hydrolysis, which is insensitive to gating affects (see methods for details). *Data (means) are representative of three or more independent experiments each performed in triplicate. Error bars represent ± standard deviation.

The high resolution of these structures allows us to model waters into the T20S (Fig. 4a). We therefore analyzed how the binding of ZYA might affect water molecules in the intersubunit pockets, that could potentially affect the conformation of the α subunits and the gate. Based on the modeled waters, we found that ZYA’s tyrosine displaced a water molecule that hydrogen bonds with the backbone of A30 and another water that further hydrogen bonds with G19 (Fig. 4b). Concurrently, we noted a water positioned to hydrogen bond with the hydroxyl group of tyrosine, and the backbone nitrogen of L21 and the side chain of E25, residues belonging to the neighboring α subunit Helix 0. To further elucidate how water contributes to the conformations we observed, we mutated E25, whose side chain interacts with only water. If E25’s role in interacting with waters is critical, we predict that E25A would perturb ZYA activation. Surprisingly, E25A T20S exhibits higher basal activity compared to WT T20S (Fig. 4c) indicating E25 helps stabilize the closed state. More importantly, neither PAN nor PA26 could activate E25A T20S. This suggests that the E25’s ability to localize specific water may be important for either regulator binding or switching to the gate-open state. These results suggest that hydration of the intersubunit pocket, or at least E25 may play important roles in 20S gating. However, the confidence of accurately identifying water molecules using cryo-EM, e.g., compared to crystallography, is not high (even at 1.9 Å), and thus other explanations could always be possible. Despite this caveat, we did consistently see the same water densities in different structures presented here (see below), and their expected displacement by ligand binding.

### Structure of T20S-L81Y demonstrates tyrosine binding plays a minor role in inducing gate-opening but can shift Helix 0, causing partial gate-opening

**Fig. 5:**
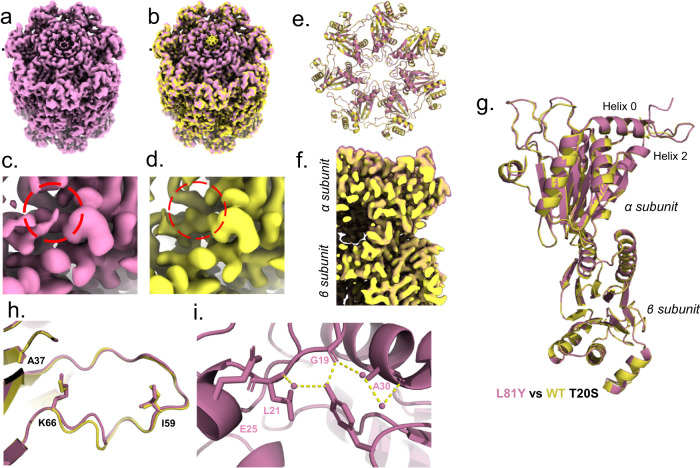
Cryo-EM structure of T20S-αL81Y (2.4 Å) mutant demonstrates partially open state by moving helix 0 without inducing HbYX-like rotations of the α subunits. **a** T20S-αL81Y unsharpened electron density map (pink). **b** Overlay of WT T20S (yellow) and T20S- αL81Y (pink) unsharpened electron density maps. **c** T20S-αL81Y electron density map (pink) of partial α ring (top view) with red dotted circle highlighting density corresponding to the position of the tyrosine of YDR motif in the open state. **d** WT T20S electron density map (yellow) of partial α ring (top view) with red dotted circle highlighting missing density corresponding to tyrosine of YDR motif. **e** Top view of atomic models showing overlay of α subunits of WT (yellow) and T20S-αL81Y (pink), aligned by β subunits. **f** Cross section of overlayed electron density maps from WT T20S (yellow-solid surface) and T20S-αL81Y (pink mesh surface). **g** Overlay of α and β subunit from atomic models of WT (yellow) and T20S-αL81Y (pink), aligned by β subunits. **h** Close-up on loop proximal to K66, with key residues discussed shown in sticks, aligned by α subunits. Coloring as in **e**. **i** Model of T20S-αL81Y (pink) showing interactions between L81Y with waters (spheres) and labeled residues (sticks).

Understanding the critical role of tyrosine in the HbYX motif, and recognizing the partial activation caused by the L81Y mutation—which was designed to mimic the tyrosine occupancy of HbYX, as identified in a concurrent study^31^—we moved forward with constructing a cryo-EM structure of T20S-αL81Y. With a resolution of 2.3 Å (PDB:8F66), this mutant allows us to perform a more thorough analysis of tyrosine’s influence on gate-opening (Supplementary Figs. 5, 6; Fig. 5a, b) (Table 1). Our EM map indicated expected densities corresponding to the open state of the YDR region in our map (Fig. 5c–red circle), which were not visible in our WT T20S map (Fig. 5d). However, compared to the ZYA-T20S map, which appear to have a fully opened gate, the YDR densities in the L81Y map were weaker (compare Figs. 1c–5c), suggesting a partially opened gate, as previously indicated by our biochemical data^31^.

To determine conformational changes induced by the single mutation mimicking the penultimate tyrosine, we compared the T20S-αL81Y model against our WT T20S (Fig. 5b, e–g; Supplementary Movie 2) and ZYA-T20S models. Unlike the ZYA-T20S model, the conformational changes caused by L81Y were different and subtle. We did not observe a substantial α-ring rotation or a conformational change at the loop proximal to K66 (Fig. 5b, e, h); however, we did observe a slight rise of Helix 0 in a direction parallel with the sevenfold axis mostly clearly seen in Fig. 5b. In fact, we noted a slight rise of the entire surface of the α subunits away from the β ring, with the most prominent changes in Helix 0 (Fig. 5b, e, f). Interestingly, alignment of individual α subunits revealed minimal intrasubunit conformational changes at this resolution. We presume the subtler effect is due to the slightly different placement of tyrosine in the L81 position compared to the tyrosine in ZYA when bound to the T20S. Similar to ZYA-T20S, the L81Y mutation caused minimal or no conformational changes in the β subunits (Fig. 5f, g).

The T20S-αL81Y model shows that the hydroxyl group of tyrosine is within proximity to hydrogen bond with the backbone of G19, similar to ZYA’s tyrosine (Fig. 5i). Additionally, we noted that tyrosine hydrogen bonded with a water molecule that is also hydrogen bonding to the backbone of L21 but not the side chain of E25 (Fig. 5i). We also noted the water which hydrogen bonds with the backbone of A30 is not displaced by L81Y, reinforcing the point that L81Y is not oriented like ZYA’s tyrosine. This structure suggests that the tyrosine interactions with G19 and L21 are sufficient to cause a Helix 0 shift upward, which consequently partially opens the gate. Therefore, the L81Y tyrosine by itself cannot fully mimic the ZYA-bound open state. This indicates that the bridging effect between G19 and K66 implemented by ZYA is likely important to induce the α subunit rotation that leads to the fully open state. Insight into how the L81Y partially induces gate opening will be further discussed below.

### Conformational changes in the N-terminus of these activated T20S structures uncover interactions that stabilize the open and closed gate conformations

While analyzing our structures to identify interactions responsible for stabilizing the gate in the open state, we noted conformational changes on the N-terminal tail in both ZYA-T20S and T20S-αL81Y models. The structure of the N-terminal tail in the closed gate conformation has not been well resolved in previously published structures of the T20S proteasome; thus, our high-resolution structures of the WT T20S (2.1 Å), ZYA-T20S (1.9 Å), and T20S-αL81Y (2.3 Å) provide insight to how the gate is stabilized in the close and open states.

**Fig. 6:**
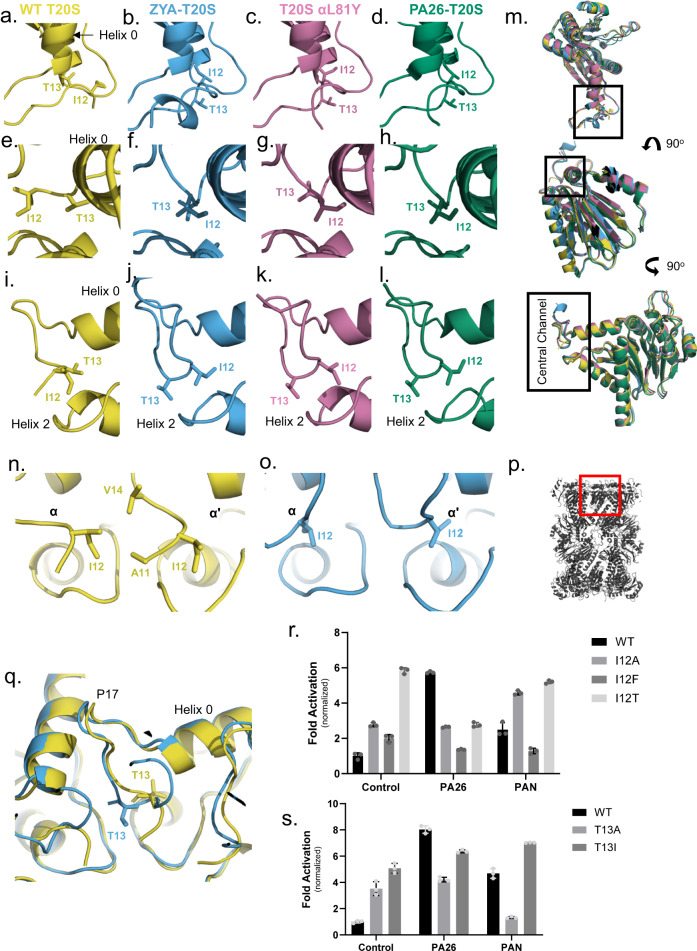
High-resolution WT T20S structure (2.1 Å) combine with ZYA-T20S structure shows αI12-αT13 play pivotal mechanistic role in switching the gate between open and closed states. **a** View of WT T20S (yellow) IT switch residues, I12 and T13 (sticks) in the closed gate state. **e** Same as A but rotated 90°. **i** Same as **e** but rotated 45°. **b**, **f**, **j** | **c**, **g**, **k** | **d**, **h**, **l** Same as **a**, **e**, **i** but for ZYA-T20S (blue), T20S-αL81Y (pink), and PA26-T20S (green), respectively. **m** (Top) View of IT Switch (box) on a single α subunit, corresponding to **a**–**d**; (middle) same as top after being rotated 90 degrees as shown, corresponding to **e**–**h**; (bottom) same as middle after being rotated 90 degrees as shown, corresponding to **I**–**l**. **n** I12 (left residue) of the IT switch in proximity to V14 and A11 (sticks) from neighboring α subunit in the WT T20S, corresponding to closed gate state. **o**. Same view as *n*, except in ZYA-T20S in the open state. I12 does not interact with neighboring α residues but instead interacts under helix 0. **p** Lengthwise cross-section view of 20S, oriented as shown in **n**, **o**. **q** Overlay of WT T20S and ZYA-T20S showing the IT switch in the closed (WT T20S, yellow) and open (ZYA-T20S, blue) states. **r** 7 nM T20S (WT, I12A, I12F, or I12T) incubated with 44 nM PA26 or 15 nM PAN (supplemented with ATP and MgCl_2_). LFP degradation rate (rfu/min) normalized as in Fig. 2e). **s** 7 nM T20S (WT, T13A, or T13I) incubated with 44 nM PA26 or 15 nM PAN (supplemented with ATP and MgCl_2_). LFP degradation rate (rfu/min) normalized as in Fig. 2e. Data (means) are representative of three or more independent experiments each performed in triplicate. Error bars represent ± standard deviation.

Our cryo-EM structure of the WT T20S now resolves more of the N-termini in the closed state, showing clear density beyond the prior resolved T13 to also show the location of I12 and A11. In this T20S model, the T13 side chain occupies the space between Helices 0 and 2 (Fig. 6a, e, i) and I12 is seen binding to a hydrophobic pocket created by the neighboring subunits N-termini comprising A11, I12, and V14 (Fig. 6n). Interestingly, the ZYA-T20S model shows that T13 is pulled out of the pocket from under Helices 0 and now I12 binds into this same pocket, which is mostly hydrophobic, containing A11, I12, and V14 of the same subunit (Fig. 6a, e, I versus b, f, j; Supplementary Movie 3). Concurrently, in the ZYA-T20S model, I12 no longer interacts with the neighboring subunits N-termini (Fig. 6o). Simply put, I12 and T13 switch binding locations under Helix 0 to switch from the closed to the open state. It appears that the ZYA-binding induced rotation of the α-subunit, that is associated with the movement of Helix 0 and displacement of P17, “pulls” T13 out of the Helix 0 pocket, and I12 closer to this pocket, allowing it to bind in this position to stabilize gate opening (Fig. 6o and Supplementary Movie 3). Going forward, we refer to the I12, T13 motif and this switching mechanism as the “IT switch”.

We next looked at I12/T13 in the structure of the T20S-αL81Y and found that it was similar to the ZYA-bound state (compare Fig. 6c, g, k to Fig. 6b, f, j; Supplementary Movie 4) indicating that mutation of L81Y also induced gate-opening via the IT switch. However, as expected, the T20S-αL81Y EM density corresponding to the two residues was not as well resolved compared to the map of ZYA-T20S (see density fit in Supplementary Fig. 7A), since the resolution of this structure was not as high, and this mutant did not generate as strongly open state as ZYA. The L81Y’s effect on the IT switch is interesting, since there is minimal displacement of Pro17, and the only significant conformational change is the rise in Helix 0. Based on this we hypothesize that the rise in Helix 0 slightly alters the IT switch binding pocket, making it more favorable to bind I12 over T13. Thus, pushing the structural equilibrium toward the open state by primarily only affecting the IT switch binding pocket.

To elucidate if the IT switch is specific to the HbYX-dependent gate opening, we compared our WT T20S model to the previously published PA26-T20S model (PDB:1YA7), which could provide insights. Interestingly, we observe a similar conformational change in the IT switch of the PA26-T20S structure (Fig. 6d, h, l; Supplementary Movie 5). It is apparent that the displacement of Pro17 allosterically triggers the switching of the “IT switch” and that the PA26-induced open conformation of the IT switch specifically is essentially identical to the ZYA bound state. The IT switch mechanism functioning to stabilize gate opening by ZYA-binding, mutation of L81Y and PA26-binding demonstrates that the IT switch is a general structural feature of the open and closed states of the T20S. However, all three mechanisms of activating the IT switch are implemented in mechanistically distinct ways, each of which affects the IT switch.

To further confirm that the IT switch plays a central role in proteasome gating, we mutated the I12 and T13 residues. Both I12 and T13 appear to play roles in both the open and closed structures. For example, in the closed state, I12 is bound to the neighboring N-termini’s hydrophobic pocket (Fig. 6n), and T13 is bound under Helix 0; then in the open state I12 binds under Helix 0 and T13 binds near V129/R130 in the other neighboring subunit (Fig. 6q). These dual roles in both states complicate effects due to mutagenesis so we first asked what the effects would be on the basal WT T20S activity. Importantly, disruption of the closed conformation could have two different effects: 1) stabilization of the open state or 2) an increase in structural disorder/entropy of the N-termini which could have a partial activation effect. Prior studies suggest the N-termini of the closed state in the T20S are disordered, but our WT structure shows a high degree of order up to residue 11 with residues 1-10 being disordered, though still occupying the central channel.

We first mutated I12 to alanine (A), phenylalanine (F), or threonine (T) (Fig. 6r). The I12A mutant is expected to reduce hydrophobic interactions with the neighboring subunits A11/V14 pocket in the closed state, and indeed this mutant was about threefold more active than WT (Fig. 6r), which is consistent with I12’s role stabilizing the closed state. The I12F had less of an effect with approximately twofold activation (Fig. 6r), which is consistent with more retention of hydrophobic interaction with its neighbor despite the added mass. The I12T mutation showed 6-fold activation (Fig. 6r), consistent with this polar residue not being supportive of the required hydrophobic interactions with the neighbors A11 and V14 residues. We next mutated the T13 residue to alanine or isoleucine (Fig. 6s). The T13A mutation again increased the basal activity of this mutant (~3.5-fold) (Fig. 6s), which would be expected with a loss of interactions with the IT switch pocket under Helix 0 due to substitution with the much smaller alanine sidechain. Similarly, the T13I mutation resulted in approximately fivefold activation (Fig. 6s). This was unexpected since isoleucine can bind to the IT switch pocket, in both the ZYA and PA26-induced open states. We hypothesize that the T13I mutation increases the interaction of the isoleucine with the neighboring V129/R130 binding pocket in the open state, rather than disrupt the closed state. Regardless, these results demonstrate that these IT switch residues are important for the maintenance of the closed state of the proteasome and its overall function.

We next determined if these IT switch mutants could be activated by either PA26 or PAN. Interestingly PA26 could not activate any of the I12 mutants we generated compared to the control, and even reduced the activity of the I12T mutant. Likewise, PAN could not activate the I12F or I12T mutants but did activate the I12A by almost twofold. First these results again demonstrate the critical function of the IT switch in regulating gate-opening by these proteasome regulators. Furthermore, since PAN can activate I12A, but PA26 cannot, this suggests that the HbYX and PA26 mechanisms are indeed distinct. It appears that the α-subunit rotation induced by HbYX-binding alters the IT switch pocket (different than PA26 does), allowing some compatibility with the I12A residue docking under Helix 0 to stabilize the open state to some extent. Since the I12F mutation showed the least activation of the T20S, and completely prevented PA26 or PAN from stimulating gate-opening, we interpret this to indicate that the phenylalanine side chain is to large and bulky to properly fit in the IT switch binding pocket, and thus prevents stabilization of the open state. The I12T mutation, however, is very activating and stimulates gate opening as well as PA26-binding does. Thus, the fact that PA26 nor PAN can further activate this mutant is not very surprising since it may be fully open in the basal state. The inhibition of this mutant by PA26 but not PAN suggests again that these regulators affect gating mechanistically in different ways. For the T13 mutants, PA26 did not stimulate T13A (p-value: 0.082) and slightly stimulated T13I, but not to the level of WT. PAN, however, significantly inhibited T13A and stimulated T13I a bit more than PA26. Thus, it would appear that the T13I does not form a stable closed structure (discussed above) and can only be stimulated to a more open state by a small amount. The T13A mutation, however, again shows different results for these two activators, with PA26 having minor impact on its activity by PAN substantially inhibiting it. This again suggests distinct roles of PAN and PA26 in how they induce gate opening. Together these data strongly support the role of the IT switch in being a central player in regulating the closed and open states of the proteasome gate.

### Mechanistic differences between HbYX-dependent (ZYA) and HbYX-independent (PA26) induced gate opening

**Fig. 7:**
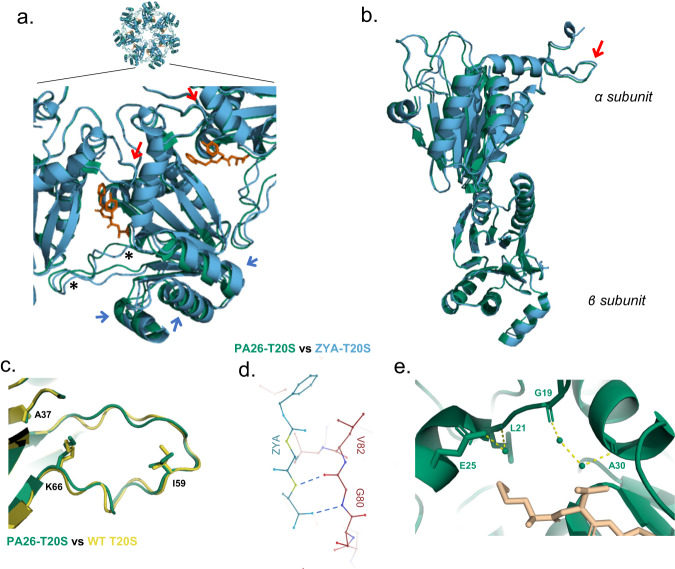
The HbYX motif (ZYA) induces gate-opening by different mechanistic principles than does PA26. **a** Top view overlay of α subunits only from PA26-T20S (green) and ZYA-T20S (blue). Red arrows show Pro17 loop. Misalignments show that different conformational changes are present in ZYA bound versus PA26-bound states, e.g., alpha helixes (blue arrow), and differences in back loops (*). **b** Overlay of PA26-T20S (green) and ZYA-T20S (blue) α and β subunits, aligned by β subunit. Red arrow shows Pro17 loop. **c** View of loop proximal to K66, including overlay of PA26-T20S (green) with WT T20S (yellow) with key residues shown in sticks. **d** ZYA forming a β-sheet-like interaction with residues, V82 and G80. **e** Model of PA26-T20S showing interactions between PA26 C-terminus with waters (spheres) and T20S α residues (sticks).

To elucidate how HbYX (i.e., ZYA)-induced gate opening differed from PA26-induced gate-opening, we compared our ZYA-T20S model against the PA26-T20S model (PDB:1YA7) (Fig. 7a, b). Both activators show key commonalities and six significant differences in the way they appear to induce gate-opening. Both ZYA and PA26 C-termini interact with the base of the intersubunit pockets via β-sheet-like H-bonding (Figs. 2d and 7d)^9^. More importantly, both ZYA and PA26 trigger conformational change in the IT switch to stabilize gate opening (Fig. 6), and both cause the Pro17 to move away from the central pore “pulling” on the IT switch. The primary difference in mechanism appears to be: (1) how the Pro17 gets moved away from the pore. When PA26 binds to T20S, its activation loop displaces Pro17, without inducing any rotation in the α subunits^9^. However, when ZYA binds, a different conformation is seen in the α ring. We overlaid the T20S α ring from the PA26-bound and ZYA-bound states to see differences in the “gate-open” states induced by these two activators (Fig. 7a, b). Notice that Pro17 is, as expected, in similar positions, however the areas of non-overlap show differences in conformation of the 20S activated states. For example, relative to PA26 binding, ZYA-binding causes the α subunits to rotate around the radial axis (Supplementary Movie 1), which is clearly visible in Fig. 7a (blue arrows). Interestingly, this ZYA-induced α rotation (combine with intrasubunit conformational changes) causes the Pro17 displacement that appears to trigger the IT switch to the open state (Fig. 1h). (2) ZYA-binding also rearranges K66 (Fig. 2b versus Fig. 7c), but PA26 binding does not appear to do this. This intriguing K66 rearrangement appears to be due to, (3) the limited length of the intersubunit “bridging” of the YX residues in the HbYX motif described above (Fig. 2c), combine with the backbone H-bond network stabilizing this position (Fig. 7d). We imagine that this lack of intersubunit “bridging” across the pocket explains why PA26 does not cause the α ring rotation that we observed in HbYX-dependent gate-opening. (4) The K66 adjacent back-loop gets rearranged by ZYA binding, but not by PA26-binding (Fig. 7a, see asterisks), which is also clearly seen by comparing Figs. 2b and 7c (presumably due to K66 reorganization (Fig. 2b). (5) ZYA-binding rearranges water molecules in the intersubunit pockets differently than does PA26-binding (Fig. 4b versus Fig. 7e). In fact, the lack of a tyrosine residue in the penultimate position meant that the C-terminus of PA26 did not displace or interact with the water molecules hydrogen bonding with the backbone of G19 and A30 (Fig. 7e). Additionally, the lack of tyrosine meant that there was no hydroxyl group present to interact with the water already hydrogen-bonding to the side chain of E25 and the backbone of L21 (Fig. 7e). Thus, the binding of the HbYX motif appears to rearrange waters in the intersubunit pocket differently than does PA26’s C-termini. These five features we observe in the ZYA-bound T20S are not seen in the PA26-bound structure, leading us to conclude that while both mechanisms converge on IT switch activation, they each trigger the IT switch in divergent ways. Collectively, these structures clearly distinguish the HbYX-dependent mechanism of activation from the PA26 mechanism, and highlight the causal interactions involved in HbYX-specific gate-opening and the associated conformational changes.

## Discussion

The impairment of the proteasome has been implicated in the etiology of NDs such as Alzheimer’s and Parkinson’s Diseases^3–7^, though the cause is unclear. In addition to regulating the proteome, studies have indicated that the function of the UPS is crucial for neuronal synapse function such as plasticity and synaptic protein turnover^1,32^, which could explain why proteasome impairment might contribute to neurodegeneration. Our prior efforts have shown specific pathological oligomers from ND directly impair the HbYX-dependent activation mechanism. Therefore, it is important to understand and elucidate the mechanisms of proteasome activation to provide avenues to potentially treat the aforementioned pathological proteinopathies. In support of this approach, a prior study of ours generated the first multicellular organism (*C. elegans*) with a constitutively open 20S proteasome that is hyperactive and resistant to oligomeric impairment^33^. These hyperactive proteasome worms presented with increased lifespan and resistance to multiple proteotoxicities, such as heat shock and oxidative damage. Studies using other means also showed feasibility and benefits of increasing proteasome amount in mammalian cells and multicellular organisms, such as increasing the degradation of endogenous and ND-related proteins^33–38^. Thus, we present the study here, motivated to explicate the mechanisms of proteasome activation.

**Fig. 8:**
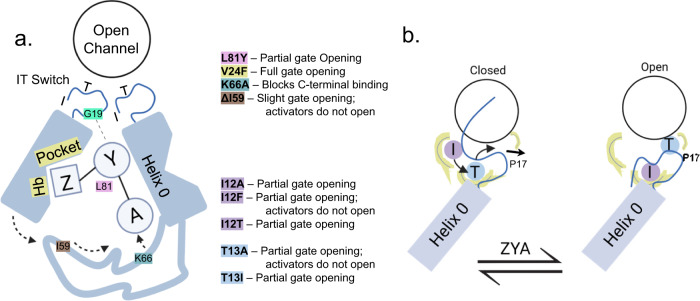
Model of critical residues and induced conformational changes involved in the molecular mechanism of gate-opening induced by a minimal HbYX motif. **a** Summary figure of HbYX motif interactions with intersubunit pocket. Color key shows residues that were mutated in this study and summarizes their effects on ZYA function. **b** Schematic demonstrating the function of the IT switch (I12, T13) that stabilizes the open and closed states of the proteasome gate. Functionally the IT switch is allosterically tethered to the Pro17 position, which is affected by the binding of ZYA and likely all proteasome activators that induce gate-opening. A color key shows residues that were mutated in this study on the IT switch and summarizes their effects on the function of the gating in the T20S proteasome.

Several cryo-EM structures of the 26S^17,18,27,28^ have been generated in the closed and open-gate states but these structures alone could not explain how the HbYX motif induces gate-opening. This is in part due to the complexity and dynamics of the interaction interface (described in the introduction) that encompasses 7 different 20S α−subunits and six different 19S ATPases subunits (RPT 1–6). To circumvent this mechanistic complexity, we first focused on the T20S from archaea, which is homoheptameric (i.e., mechanistically simpler), and contains the same conserved structural gating elements (e.g., N-terminal YDR motif, Pro17-reverse turn, closed and open states). The 1.9 Å cryo-EM structure of ZYA bound to the T20S also showed that it bound to the intersubunit pockets precisely the same way as the HbYX motif, establishing ZYA as a minimal HbYX mimicking compound. We further probed all three sub-binding pockets of ZYA to determine the potential mechanistic contributions of the Hb, Y, and X positions, in inducing gate opening. Combining the results from the mutagenesis and structures allowed us to develop a mechanistic model for how the HbYX motif (or ZYA) is able to induce gate opening and uncover an important component of the gating function—the IT switch, whose function is summarized in Fig. 8.

While the IT switch is clearly important in archaea proteasome, does it play a role in regulating the H20S? To answer this question, we compared the sequence of the T20S to the 7 different H20S α subunits and determined if similar IT switch function was conserved in the H20S and H26S proteasome. We found that indeed there is a high conservation of the IT switch motif (e.g., a hydrophobic residue paired with a polar residue) in all α subunits except α3, which instead has a conserved pair of threonine residues (i.e., “TT” instead of “IT”; Supplementary Fig. 7b). In addition, this IT motif is separated from the critical YDR motif by exactly one residue in T20S and all seven human α subunits. Additionally, the critical Pro17 is 3 residues away from the IT switch in all cases. Interestingly, we found that α4’s N-terminus is far more conserved with the N-termini of the T20S α subunit then any of the other human α N-termini (Supplementary Fig. 7c, d), and correspondingly that α4’s IT switch is identically conserved with I and T residues. This is noteworthy because the T20S α N-termini all participate in gate closing and opening, since it is a homoheptamer, while primarily the N-termini of the heterohexameric α2, 3, and 4 subunits in the H20S participate in gating. Together this conservation of the IT switch in H20S, and particularly the strong conservation in the α 4 N-termini, which plays a significant role in the closed gate, suggests that it may play a key role in regulating gate-opening in the H20S.

To assess the potential functional role of the IT switch in H20S gating, we analyzed structural changes that occur upon gate-opening in the 26S proteasome when the 20S is closed (E_A1_) and open (E_D2_;)^17^ states (PDB: 6MSB and 6MSK). The N-termini of α’s 2, 3, and 4, are the primary ones that undergo opening and closing, although α1 and α5 do make small conformational changes and contribute to the closed state. We found that the IT switch functions in α2 (LT) and α4 (IT) the same way it does in the T20S—with the “T” under Helix 0 in the closed state and switching to the hydrophobic residue (I or L) under Helix 0 in the open state (summarized interactions in Supplementary Fig. 7e, f). This indicates the IT switch is indeed functionally conserved in the H20S. Interestingly, the residues corresponding to the IT switch on α3 (TT) function a bit differently. The α3 T10 residue is out of the Helix 0 pocket in the closed and open states (Supplementary Fig. 7f) but the α3 T9 side chain function similarly to the I12 in the T20S and moves into the pocket under Helix 0 when the 20S is activated. When we mutated the homoheptameric T20S IT switch to resemble the IT switch of α3 (i.e., T20S-I12T to make a “TT” pair), this mutation appeared to destabilize I12 interaction with its neighbor in the closed state. However, the α3 T9’s neighbor in the closed state does not present a hydrophobic pocket, but rather another threonine (α2-T12), seemingly promoting a stable closed state with a T in this normally hydrophobic IT switch position. As for α6 and 7, they are always in the open state, and both of their hydrophobic IT switch residues stay in the position under Helix 0 as expected for IT switch function. Lastly, α1 and 5 both interact, to a small degree, with the α2, 3, 4 closed state and their N-termini do move to a more “open” state upon activation. Consistent with this, α5’s hydrophobic IT switch residue moves in under Helix 0 in the open state also consistent with IT switch function. However, α1’s IT switch is a clear outlier, its T14 IT switch residue (at least in these models) stays in place under Helix 0 in both states. In conclusion, the IT switch function uncovered in the T20S is well conserved in sequence and presumably function in the H20S for 6 of the 7 αsubunits, especially when focusing on the role of the hydrophobic residue switching to the Helix 0 pocket, as we found in the T20S.

By comparing the structures of WT T20S (with extended resolved N-terminal residues), with ZYA-T20S, T20S-αL81Y, and previously published PA26-T20S, we develop a detailed molecular model of how the HbYX motif induce gate opening without contacting Pro17 (as PA26 does with its activation loops^9^). In this model, the HbYX Tyrosine-Alanine connects two neighboring α subunits by its tyrosine H-bonding to G19 and alanine carboxy salt bridging with K66 in the neighboring α subunit. While this interaction has been noted previously, at this higher resolution we observe a shift in the K66 side chain (Fig. 2b), its stabilization by a network of ionic interactions (Fig. 2d), and a rearrangement of this loop at the back of the intersubunit pockets (Fig. 2b, d; D57-I67 or “back-loop”) which is naturally more mobile (Supplementary Figs. 2, 4). The length of this “bridge” appears to be critical for gate-opening as ZYA analogs that lengthen the “YA” bridge (e.g., Z-phospho-YA) are inactive^31^, which supports the model that this back-loop reconfiguration is important for gate opening. Moreover, K66 and a back-loop of proper length (Fig. 2e) are required for gate-opening, as the tyrosine by itself, T20S-αL81Y, did not induce full gate-opening or α subunit rotations. Combined, these indicate an important role of the “bridge” and the “back-loop” for inducing α subunit rotations that cause gate-opening in the ZYA-bound state.

It is our working model that the reorganization of the back-loop, which allows for the neighboring Helix 0 to rotate towards its neighbor, sets off an allosteric chain reaction around the α ring. When Helix 0 rotates away from its neighbor’s N-termini, it causes the neighboring Pro17 to also move towards this helix to maintain its packing interaction, leading to both inter and intrasubunit-induced conformational changes in the Pro17 position (Figs. 2h and 7b). The repositioning of Pro17 then displaces the IT switch T13, pulling it out of the IT switch binding pocket under Helix 0. This T13 displacement then leaves the binding pocket empty allowing the more hydrophobic I12 to now bind under Helix 0, which reconfigures the gate and positions the N-termini into a compatible position for the YDR motif to stabilize the open state. In fact, this 1.9 Å ZYA-T20S structure now shows the open state resolved up to G4.

An additional layer to this “back-loop” rearrangement model should also include the importance of the bulky benzene group (Z) in ZYA. Mutations that placed a bulky aromatic in the Hb binding pocket (i.e., V24F, A154F, and V24Y; Fig. 3b–d) all resulted in substantial gate-opening, especially V24F. These mutants suggest that HbYX motifs with a bulky Hb position may be strong activators of T20S gate-opening. However, a high-resolution structure of one of these mutants will be needed to determine the mechanism of gate-opening by aromatic ring occupancy in the Hb binding pocket. V24F could induce gate-opening by changing the Helix 0 position, as we saw with the αL81Y mutation, or it could somehow induce α subunit rotations like ZYA does. Further study is needed to confirm the contribution to gating by the Hb pocket, though comparing activation by LYA versus ZYA provides further insight. Both small peptides could induce gate opening in the T20S^31^, but LYA presents a non-bulky aliphatic side chain to the Hb binding pocket, while ZYA presents a bulky aromatic. The fact that LYA could induce gate-opening similar to ZYA (though at lower affinity) indicates that perhaps intersubunit bridging by the YA is sufficient to induce gate-opening. However, providing a bulkier Hb group increases affinity and likely efficacy, which was also a topic in a recent structural study from the Gestwicki group that used a chimeric PA26-HbYX complex^23^. Taken all together, the Hb group likely plays an important role in increasing affinity for the intersubunit pocket but likely also plays a direct mechanistic role in helping induce gate-opening. Therefore, the effects of the Y-A bridge that reorganize the back-loop and the interaction of Hb group under Helix 0 (especially, if a bulky aromatic) combine into a minimal motif that can robustly induce proteasome gate opening in 20S proteasomes from archaea, yeast, and mammals.

Further structural studies will need to be done to determine how ZYA-induced gate opening occurs in the mammalian 20S, which has seven different intersubunit pockets. Our analysis of the 3–4 Å H26S cryo-EM structures in the open and closed states shows strong sequence and functional conservation of the IT switch mechanism (mapped out in Supplementary Fig. 7). Since the HbYX is conserved and ZYA functions similarly in T20S, Y20S, and M20S, we expect that similar mechanisms will be at play, though in a more asymmetric manner since primarily only α2, α3 and α4 contribute to the closed state, while the others are open. The H19S has been observed to engage with the H20S using all its three HbYX motifs on Rpt-2, -3, and -5; yet in the inactive 26S state the gate is observed as closed. Building on our current model, we speculate that this may be because HbYX subunits Rpt3, and 5 interact with α subunits that have their N-terminal tails already in a constitutively open conformation (α1 and α5). What about Rpt2, which interacts with α3? α3 has a modified IT switch (TT instead of IT) and the I12T mutation in T20S (TT) was more open than WT but couldn’t be activated by PA26 or PAN. This is consistent with α3 whose IT switch is not engaged under Helix 0 stabilizing its closed state; instead, α3’s N-termini is stabilized by sitting on top of and interacting with the closed N-termini of α2 and α4 (Supplementary Fig. 7f)^17^. Based on this unique α3 IT switch, and N-terminal position in the closed state, it is perhaps not surprising that α3 appears to be desensitized to HbYX binding, explaining the closed state of the inactive 26S. Conversely, Rpt1 binding to α4 in the activated H26S state (Supplementary Fig. 7) has been linked with gate opening^17^, and it carries a partial HbYX motif (-TYN). Interestingly, α4 has a perfectly conserved IT switch, and its N-terminal 1–34 residues are uncannily highly conserved relative to the T20S α subunit, more so than any other H20S α subunit’s N-termini (Supplementary Fig. 7c). These two conserved elements of α4s gating region suggest unique mechanistic importance for α4 and Rpt1 in controlling gate-opening in the eukaryotic 20S. Perhaps Rpt1s effect on the α4 subunit alone is sufficient to trigger α4s IT switch, also affecting α3 and 2, leading to gate opening, or perhaps contributions from other HbYX motifs are needed to trigger an allosteric system. Further study is needed to test this hypothesis based on the findings presented here. Nevertheless, it is apparent that the eukaryotic 26S gating system evolved a spectrum of Rpt C-termini sequences, IT switches, and α N-termini to fine-tune how gate opening is controlled by substrate binding to the 26S proteasome. We expect that the identification and function of the IT switch and the other HbYX relevant mechanism defined here for the T20S will guide understanding of how these mechanisms regulate the more complicated 26S proteasome as suggested here.

Our collective findings and analyses in this study underscore the differences and similarities in the mechanisms of proteasome activation that are either dependent or independent of the HbYX motif. Both classes of proteasome activators target the intersubunit pockets for binding and induce a relocation of Pro17 to actuate the IT switch and cause gate opening. However, they distinctly vary in the way Pro17 is relocated. Specifically, the HbYX-independent activators directly interact with Pro17 to induce its movement, while HbYX-dependent activators initiate an allosteric rearrangement within and across the α subunits. This rearrangement includes the entire α ring and results in the relocation of Pro17, actuating the IT switch. An intriguing evolutionary enigma arises from the comparison of these two activation mechanisms. It appears that different families of proteasome activators have evolved to utilize distinct methods to initiate a common mechanism within the IT switch, leading to the opening of the 20S gate.

Interestingly the mechanisms uncovered here also shed light on how oligomers could impair the 20S proteasome, without being able to enter the internal chamber of the proteasome^39^. If the HbYX mechanism requires a functioning back-loop for activation, which is supported by our structure and the activity of the T20S-I59Δ, then this back-loop could be a target for toxic oligomers that impair proteasome function by blocking HbYX-dependent gate-opening but not PA26-induced gate-opening, which we observe biochemicaly^39^. Elucidating the mechanisms of proteasomal gate-regulation such as performed in this study is critical in the development of small molecules that reverse proteasome impairment (associated with neurodegeneration) and stimulate unstructured protein degradation.

## Methods

### Proteasome purifications

*T.acidophilum* wild type (WT) 20S, Δα_2-12,_ and all other mutant 20S proteasomes were similarly purified as described^40^, except via 8XHis tags on the C-terminus of β subunit. All 20S mutants were generated by overlapping PCR site-directed mutagenesis.

### Proteasome activity assays—peptide substrates

Fluorogenic substrate peptides were purchased from BostonBiochem (suc-LLVY-amc) and EZBiolabs (LFP (Mca-AKVYPYPME-Dpa (Dnp)-amide)), ZYA was synthesized by ABclonal. Peptides were dissolved in DMSO and incubated with proteasomes at indicated concentrations. The final concentration of DMSO in activity assays was 2%. Protein concentrations were determined by Bradford assay (Thermo Scientific). To measure peptide hydrolysis, fluorogenic peptides dissolved in DMSO were used at a final concentration of 25–100 μM for Suc-LLVY-amc and 3–10 μM for LFP, in 50 mM Tris (pH 7.5), 1 mM DTT. For archaeal 20S experiments the indicated concentrations of T20S and LFP peptide was added to the buffer at 45 °C, and where not indicated, 1 μg of PAN and 10 μM ATPγS (+5 mM MgCl_2_), or 2 mM ATP and 10 mM MgCl_2_, was added to the 0.1 ml of reaction buffer (sufficient to saturate the 20S particles). Amount of archaeal 20S from different purification preparations is normalized to their rate of LLVY-AMC hydrolysis, whose degradation is not regulated by gating. Assays were performed for 30 mins to an hour and analyzed using BioTek Gen5 Data Analysis software. Activity was measured as relative fluorescence units/minute (rfu/min), generating a curve that was used to calculate the initial velocity, according to the slope of the curves. Where indicated “Fold Activation”, fold activation was calculated by dividing the average initial velocity against the average of negative controls, with added DMSO when appropriate.

### Cryo-EM sample preparation and data collection

Copper Quantifoil R 1.2/1.3 300 mesh (EMS) grids were cleaned using a PELCO easiGlow Glow Discharge cleaning system. A volume of 3 μL of 0.5 mg/mL WT T20S, T20S-αL81Y or T20S with 4 mM ZYA (suspended in 50 mM Tris pH 7.4, 150 mM NaCl) sample was placed onto a grid, and then flash frozen in liquid ethane using a manual plunge freeze apparatus. Data collection was done using a Titan Krios transmission electron microscope (Thermo Fisher) operated at 300 kW and a magnification of ×81,000, which resulted in 0.503 Å/px. Images were collected using a Falcon IIIEC direct electron detector camera equipped with a K3/GIF operating in counting and super resolution modes. Electron dose per pixel of 50 e-/Å2 was saved as 40 frame movies within a target defocus range of −2.5 to −1.25. All the data was collected using cryoSPARC software (Structura Biotechnology Inc.)^41^.

### Cryo-EM single-particle analysis

Cryo-EM images of the WT, T20S-αL81Y, and ZYA-T20S proteasome were analyzed using cryoSPARC. Schematic for cryo-EM single-particle data processing available in Supplementary Materials (Supplementary Figs. 1–6).

WT T20S: From 1744 movies collected, we picked 444,678 particles after four rounds of 2D classification, which were used to generate an Ab-initio model and processed through heterogenous refinement and then homogenous refinement (using D7 symmetry).

T20S-αL81Y: From the 2850 movies collected, we used 2847 in analysis and picked 889,069 particles after three rounds of 2D classification to obtain the best particle sets. The particles chosen from 2D classification were used to generate an Ab-initio model, which was used for homogeneous refinement (using D7 symmetry).

ZYA-T20S: From movies collect, we used 830,572 particles after two rounds of 2D classification. Particles isolated were used to generate an Ab-initio model, processed in a heterogenous refinement, then homogenous refinement (using D7 symmetry).

Final map was imported into Phenix^42^ to run density modification (DenMod) from two half maps. All representations (figures and movies) of the T20S proteasome complex were created using PyMol 2.5.2, WinCoot 0.9.6 EL, and UCSF ChimeraX v1.3^43,44^.

### Atomic model building

The atomic models were built using a modified version of the T20S from PDB: 1YA7 as a template, rigid body fitting into the electron density map using PHENIX 1.19.2-4158. The docked models were subjected to a cycle of morphing and simulated annealing, five real-space refinement macrocycles with atomic displacement parameters, secondary structure restraints, and local grid searched in PHENIX. Consequently, the models were refined by oscillating between manual real-space refinement in WinCoot 0.9.6 EL and real-space refinement in PHENIX (five macrocycles, without morphing and simulated annealing). ZYA was docked using LigandFit on PHENIX. Waters were added to models using PHENIX Douse.

### Statistics and reproducibility

Data were analyzed in Graph Pad or excel using an unpaired Student’s t-test (Prism). For all statistical analyses, a value of *p* < 0.05 was considered significant.

### Reporting summary

Further information on research design is available in the Nature Portfolio Reporting Summary linked to this article.

## Supplementary information

Supplementary Information

Description of Additional Supplementary Files

Supplementary Movie 1

Supplementary Movie 2

Supplementary Movie 3

Supplementary Movie 4

Supplementary Movie 5

Supplementary Data 1

Reporting Summary

## Data Availability

Cryo-EM maps are deposited in the Electron Microscopy Data Bank (EMDB) under accession codes, EMD-28906 (ZYA-T20S), EMD-28878 (WT T20S), and EMD-28876 (T20S-αL81Y). Coordinates are available from the RCSB Protein Data Bank under accession codes, 8F7K (ZYA-T20S), 8F6A (WT T20S), and 8F66 (T20S-αL81Y). The authors declare that data supporting the findings of this study are available within the paper and its supplementary information files and are available from the corresponding author upon request. Source data for all graphs, including individual data points, can be found in an Excel file titled Supplementary Data.
