# Supplementary material for: High resolution structures define divergent and convergent mechanisms of archaeal proteasome activation
Source: Commun Biol. 2023 Jul 15;6:733. doi: 10.1038/s42003-023-05123-3 (PMC10349882; doi:10.1038/s42003-023-05123-3)
Supplement: Supplementary file 2 — Description of Additional Supplementary Files [file 42003_2023_5123_MOESM2_ESM.pdf]

Description of Additional Supplementary Files

File name: Supplementary Data
Description: Source data includes excel file with tabs for each figure in manuscript. Source data for each figure includes individual data for enzymatic rates output by the Biotek Synergy plate reader.

File name: Supplementary Movie 1
Description: Top view of morph between WT T20S and ZYA-T20S models, only showing α subunits.

File name: Supplementary Movie 2
Description: Top view of morph between WT T20S and T20S-L81Y models, only showing α subunits.

File name: Supplementary Movie 3
Description: View of IT Switch morph between WT T20S and ZYA-T20S models.

File name: Supplementary Movie 4
Description: View of IT Switch morph between WT T20S and T20S-L8Y1 models.

File name: Supplementary Movie 5
Description: View of IT Switch morph between WT T20S and PA26-T20S (PDB:1YA7) models.
